# Impact of radiographic honeycombing on transplant free survival and efficacy of immunosuppression in fibrotic hypersensitivity pneumonitis

**DOI:** 10.1186/s12890-023-02523-3

**Published:** 2023-06-22

**Authors:** Traci N. Adams, Kiran Batra, Margaret Kypreos, Craig S. Glazer

**Affiliations:** 1 Division of Pulmonary and Critical Care Medicine, University of Texas Southwestern Medical Center, 5323 Harry Hines Blvd, Dallas, TX 75219 USA; 2 Department of Radiology, University of Texas Southwestern Medical Center, 5323 Harry Hines Blvd, Dallas, TX 75219 USA

**Keywords:** Hypersensitivity pneumonitis, Usual interstitial pneumonia, Interstitial lung disease

## Abstract

**Background:**

The distinction between hypersensitivity pneumonitis (HP) and idiopathic pulmonary fibrosis (IPF) was thought to be important due to the difference in mortality between the conditions as well as the response to treatment. However, recent work suggests that the clinical diagnosis may matter less than certain radiographic features, namely usual interstitial pneumonia (UIP) pattern. The purpose of this study is to evaluate whether radiographic honeycombing is more predictive of transplant-free survival (TFS) than other clinical, radiographic, or histologic findings that distinguish HP from IPF in the current guidelines and to evaluate the impact of radiographic honeycombing on the efficacy of immunosuppression in fibrotic HP.

**Methods:**

We retrospectively identified IPF and fibrotic HP patients evaluated between 2003 and 2019. Univariable and multivariable logistic regression was performed for patients with fibrotic HP and IPF to evaluate TFS. To assess the impact of treatment with immunosuppression on TFS in fibrotic HP, a cox proportional hazard model adjusted for known predictors of survival in HP including age, gender, and baseline pulmonary function testing results was constructed, and p-interaction for the presence of honeycombing on high resolution computed tomography and use of immunosuppression was calculated.

**Results:**

Our cohort included 178 with IPF and 198 with fibrotic HP. In a multivariable analysis, the presence of honeycombing had a greater impact on the TFS than the diagnosis of HP vs. IPF. Among the criteria used in the HP diagnostic guidelines, only typical HP scan impacted survival in a multivariable model, while identification of antigen and surgical lung biopsy findings had no impact on survival. We identified a trend toward worse survival on immunosuppression in those with HP with radiographic honeycombing.

**Conclusion:**

Our data suggests that honeycombing and baseline pulmonary function testing have a greater impact on TFS than the clinical diagnosis of IPF vs. fibrotic HP and that radiographic honeycombing is a predictor of poor TFS in fibrotic HP. We suggest that invasive diagnostic testing including surgical lung biopsy may not be useful in predicting mortality in HP patients with honeycombing and may potentially increase risk of immunosuppression.

**Supplementary Information:**

The online version contains supplementary material available at 10.1186/s12890-023-02523-3.

## Introduction

Hypersensitivity pneumonitis (HP) is an interstitial lung disease (ILD) that results from exposure to inhaled antigens [1, 2]. Making an accurate diagnosis of HP is important because, in contrast to idiopathic pulmonary fibrosis (IPF), HP has a better prognosis and can be treated with immunosuppressive medications in addition to an antifibrotic [3, 4]. Recent clinical guidelines on the diagnosis of HP rely on a combination of radiographic findings, identified antigen exposure, bronchoalveolar lavage (BAL) lymphocyte percentage, and histopathologic findings to determine the probability of HP in a patient with ILD [1]. In the guidelines, patients are categorized in order of increasing diagnostic confidence as HP not excluded, low probability, moderate probability, high probability, or definite probability of HP [1]. Integration of the IPF and HP clinical guidelines suggest the use of clinical history, radiographic assessment, and, when necessary, histopathologic assessment to distinguish HP from IPF and to estimate the diagnostic confidence [3]..

Historically, the distinction between HP and IPF was thought to be important due to the difference in mortality between the conditions as well as the response to treatment, with IPF being treated with antifibrotics and HP with immunosuppression [1, 4]. However, recent work suggests that the clinical diagnosis may matter less than certain radiographic features, namely usual interstitial pneumonia (UIP) pattern, which is a radiographic pattern defined by traction bronchiectasis, reticulations, and honeycombing in the absence of other features [1, 5–7].

In order to more fully integrate the HP diagnostic guidelines within what is now known about UIP pattern, we sought to evaluate whether transplant-free survival was more impacted by known predictors of mortality including UIP pattern, age, and baseline pulmonary function testing, or by diagnostic classification in a retrospective cohort of fibrotic HP and IPF [6]. In this study, we evaluated the impact of radiographic findings, identified antigen exposure, BAL lymphocyte percentage, and histopathologic findings on TFS in HP. We hypothesized that honeycombing would be more predictive of TFS than other clinical, radiographic, or histologic findings, and that honeycombing would impact the effect of immunosuppression on TFS in fibrotic HP.

## Methods

We retrospectively identified ILD patients evaluated between 2003 and 2019 from the University of Texas Southwestern Medical Center (UTSW). Patients were identified using an EPIC registry of patients with a diagnosis of ILD or a subtype of ILD seen in our ILD clinic. This study was conducted in accordance with the amended Declaration of Helsinki, and the UTSW Institutional Review Board approved the study and waived consent given the retrospective nature of the study (STU-2019-0913). Patients who had a multidisciplinary diagnosis of fibrotic HP or IPF were included in the study. At our center, multidisciplinary discussions includes our thoracic radiologist (author KB), pathologist, and multiple pulmonologists (authors CSG, MK, TNA). Hypersensitivity pneumonitis exposures are assessed using a standardized template. Connective tissue disease is excluded by history, physical examination, and connective tissue disease serologies; rheumatology consultation is obtained when there is clinical suspicion for autoimmune disease. A repeat multidisciplinary discussion including re-review of pathology was not conducted for all patients for the purposes of this study; however, high resolution computed tomography (HRCT) which included inspiratory, expiratory, prone, and supine images, was reviewed again for this study by thoracic radiologist (KB) who was blinded to the clinical diagnosis. In addition, the medical record of each patient included in the study was reviewed to ensure that the diagnosis had not changed (including the development of connective tissue disease or explant pathology that suggested an alternative diagnosis) and the descriptions of histopathologic findings from the initial multidisciplinary meeting were used for this study. Patients with nonfibrotic HP were excluded from the analysis, as nonfibrotic HP is a distinct phenotype known to have a substantially better survival than fibrotic HP [1, 2, 6]. The correlation between our radiologist’s high resolution computed tomography (HRCT) interpretations with that of radiologists from other academic medical centers has been previously established in the literature [8]..

The diagnosis of HP and the level of diagnostic confidence was determined by the American Thoracic Society guidelines [1]. Patients were classified as HP if they had a moderate, high, or definite probability of HP. Exposure history was reviewed by an occupational medicine specialist (CSG) to determine whether the exposure was significant enough to lead to sensitization [2]. BAL lymphocytosis was defined as greater than 30% lymphocytes. HRCT images were classified as indeterminate, compatible, or typical HP according to current guidelines [1]. The presence of honeycombing and UIP pattern was also noted [4]. Surgical lung biopsy (SLB) findings were classified as indeterminate, probable, or definite HP according to current guidelines [1]..

Clinical data extracted from the medical record included age, gender, ethnicity, smoking history, potential antigen exposure, HRCT features, pulmonary function testing (PFTs), bronchoalveolar lavage lymphocytosis, antifibrotic use, and histopathologic sampling via transbronchial biopsy and SLB. The use of immunosuppression was also extracted from the medical record, and patients were considered to be on immunosuppression if they had > 6 months of continuous use of mycophenolate, azathioprine, and/or prednisone.

Continuous variables were expressed as means and standard deviations, and comparisons were made using Student’s t test or Wilcoxon signed rank sum test. Categorical variables were expressed using counts and percentages; comparisons were made using Chi-squared test or Fisher’s exact test, where appropriate. Univariable logistic regression was performed for patients with fibrotic HP and IPF to evaluate transplant free survival. Variables included known prognostic factors for HP including demographic and physiologic variables as well as radiographic features, antigen exposure, diagnostic confidence, and histopathologic findings. The variables that were significantly associated with change in diagnosis (p-value < 0.1) were included in multivariable model to test independent associations. The multivariable model was run separately with the criteria for diagnosis and the overall diagnostic confidence levels, as there is a significant relationship between diagnostic confidence and the variables used to classify that confidence. To assess the impact of treatment with immunosuppression on TFS in fibrotic HP, a cox proportional hazard model adjusted for known predictors of survival in HP including age, gender, forced vital capacity (FVC) % predicted, and diffusion capacity for carbon monoxide (DLCO) % predicted was constructed, and p-interaction for the presence of honeycombing on HRCT and use of immunosuppression was calculated. All p-values less than 0.05 were considered significant.

## Results

**Fig. 1:**
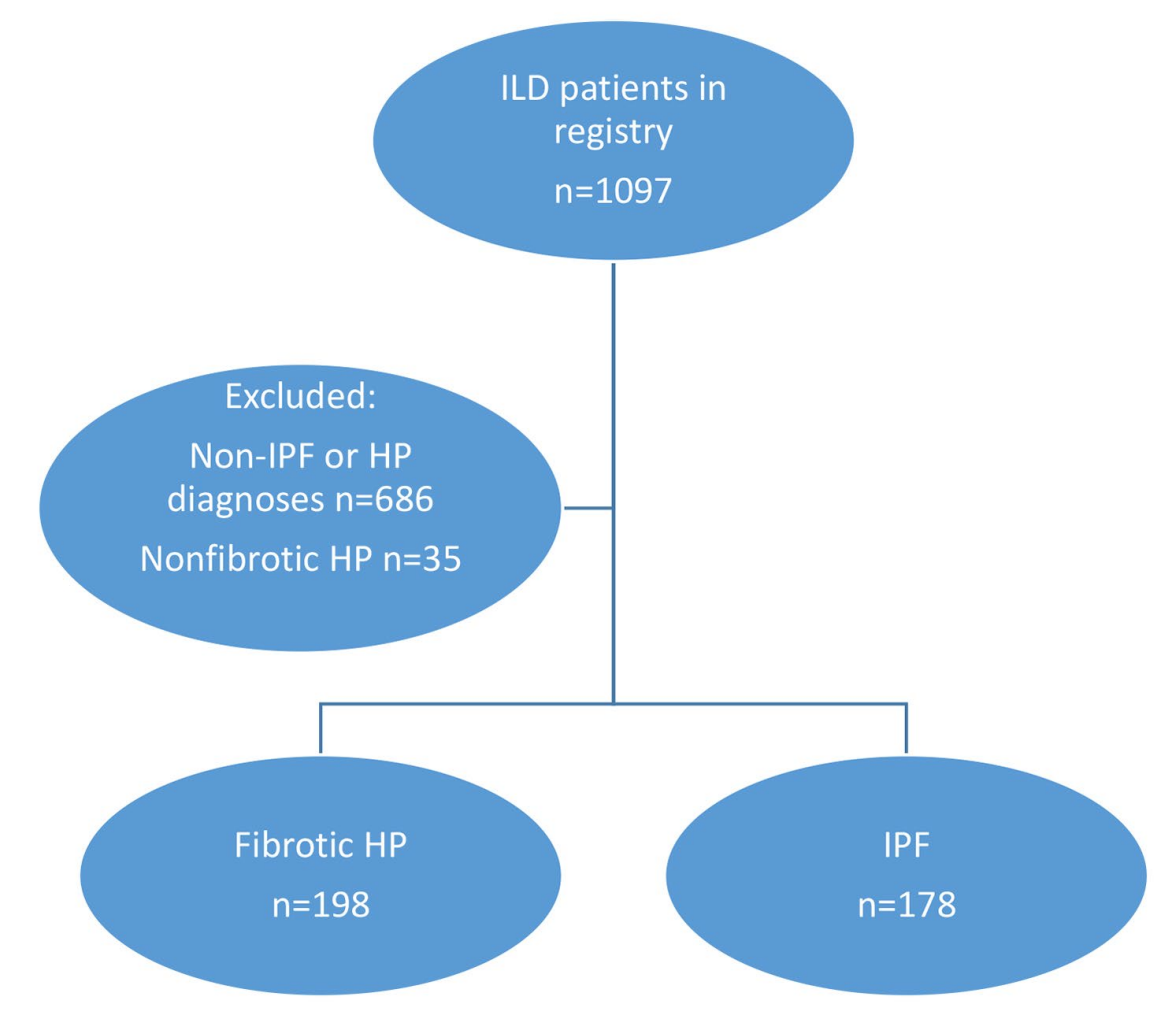
Flow diagram of the study cohort

Our cohort contained 1097 patients. Of these, 178 had IPF and 198 had fibrotic HP based on published criteria and multidisciplinary discussion and were included in the study (Fig. 1). Demographic, clinical, radiographic, and histopathologic characteristics of the cohort are available in Table 1. All patients on immunosuppression had a dose of mycophenolate of 2 g daily or higher or azathioprine 100 mg per day or higher, and when prednisone was used as monotherapy all patients were on doses between 10 mg and 40 mg.

**Table 1:** Characteristics of the cohort.

	HP cohort (N = 198)	IPF cohort (N = 178)
Mean age (SD)	64.6 (10.0)	68.2 (8.6)
Male, No. (%)	99 (50.0)	131 (73.6)
Ethnicity, No. (%)		
Non-Hispanic White	162 (81.8)	133 (74.7)
Black	4 (2.0)	7 (3.9)
Hispanic or Latino	16 (8.1)	21 (11.8)
Asian	6 (3.0)	3 (1.7)
Unknown	6 (3.0)	14 (7.9)
Ever Smoker, N (%)	98 (49.5)	112 (62.9)
Antigen identified, No. (%) *	172 (86.7)	50 (28.1)
Mold antigen	80 (40.4)	26 (14.6)
Avian antigen	97 (49.0)	39 (21.9)
Other**	21 (10.6)	0 (0.0)
Baseline Lung Function, mean (SD)		
FVC % predicted, N	66.3 (18.2), 194	74.4 (20.6), 178
DLCO % predicted, N	49.1 (17.3), 188	52.0 (22.5), 173
HRCT available for scoring	198 (100)	178 (100)
Typical HP	116 (58.6)	0 (0)
Compatible HP	24 (12.1)	23 (12.9)
Indeterminate HP	58 (29.2)	154 (86.5)
Honeycombing on HRCT	61 (30.8)	94 (52.8)
Definite UIP pattern	10 (5.1)	78 (43.8)
BAL lymph performed	63 (31.8)	18 (10.1)
BAL lymph > 30%	24 (12.1)	3 (1.7)
SLB available for scoring	129 (65.2)	49 (27.5)
Typical HP	56 (28.3)	0 (0)
Compatible HP	59 (29.8)	0 (0)
Indeterminate HP	14 (7.1)	49 (27.5)
Diagnostic confidence HP		
Definite HP	85 (42.3)	0 (0)
High probability HP	31 (15.7)	0 (0)
Moderate probability HP	82 (41.4)	0 (0)
HP low probability or not excluded	0 (0)	178 (100)
Antifibrotic use for > 6 months	10 (5.1)	160 (89.9)
Death or transplant	58 (29.3)	103 (57.9)

*Some patients had more than one identified antigen

**Other antigens include isocyanate exposure and fish tank exposure

**Fig. 2:**
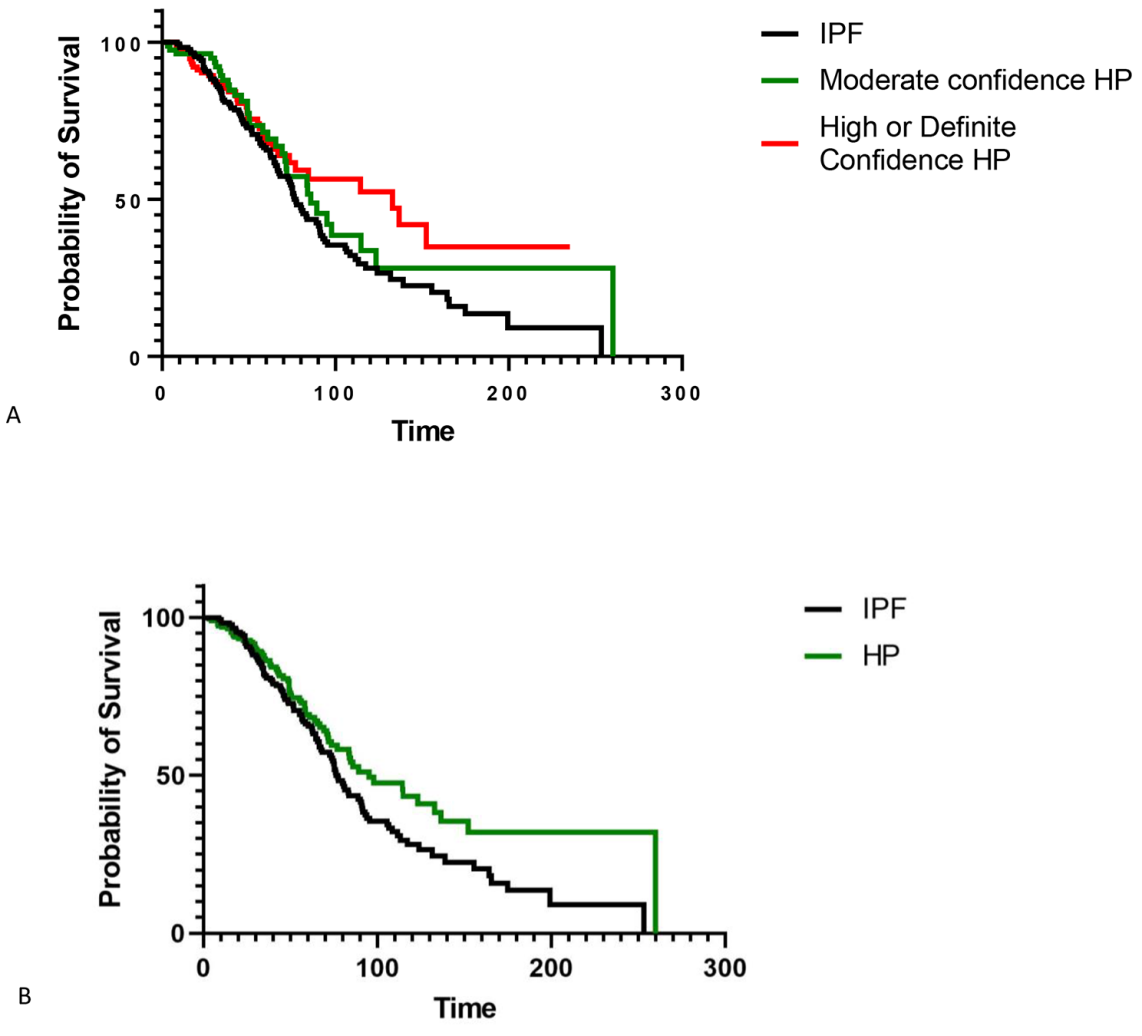
**A** Survival of hypersensitivity pneumonitis stratified by diagnostic confidence zcompared to idiopathic pulmonary fibrosis; Fig. 2B. Survival of hypersensitivity pneumonitis compared to idiopathic pulmonary fibrosis. IPF = idiopathic pulmonary fibrosis; HP = hypersensitivity pneumonitis

**Fig. 3:**
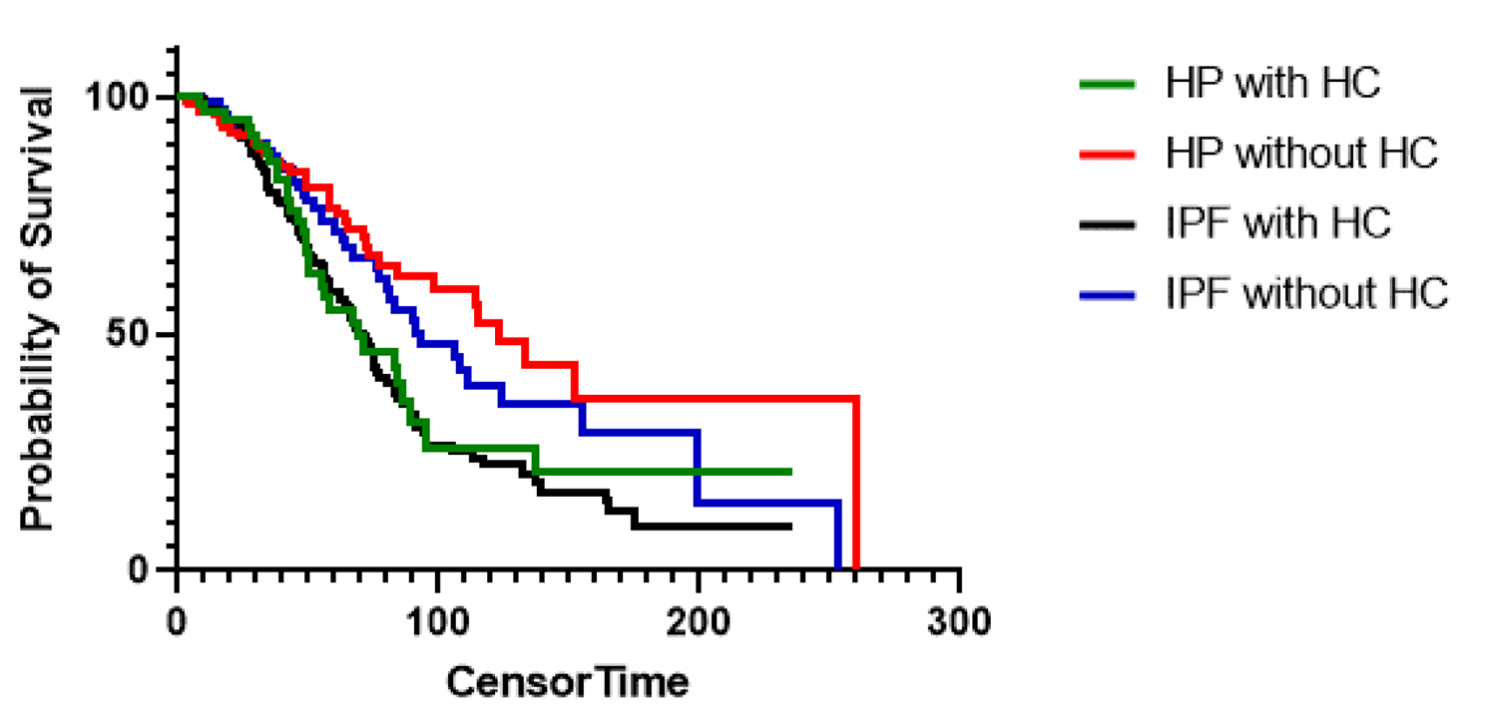
Survival of hypersensitivity pneumonitis with and without radiographic honeycombing compared to idiopathic pulmonary fibrosis. HC = honeycombing; HP = hypersensitivity pneumonitis; IPF = idiopathic pulmonary fibrosis

Among patients with fibrotic HP and IPF, honeycombing was a predictor of transplant free survival in a univariable (HR 1.52, 95% CI 1.13–2.05, p = 0.006) and multivariable model (HR 1.64, 95% CI 1.2–2.26, p = 0.002) adjusted for age, gender, FVC % predicted, DLCO % predicted, antigen identification, and diagnosis (Table 2). IPF had a worse survival compared to high or definite confidence HP in a univariable model (HR 1.46, 95% CI 1.02–2.15, p = 0.045) but this did not persist in a multivariable model (HR 1.3, 95% CI 0.84–2.03, p = 0.26) (also shown in Fig. 2A). There was no difference between transplant free survival in IPF and moderately confident HP in a univariable or multivariable model. When all HP vs. IPF was compared without stratifying by diagnostic confidence of HP, IPF had a worse survival than HP in a univariable (HR for IPF 1.4, 95% CI 1.03–1.90, p = 0.03) and multivariable model (HR for IPF 1.45, 95% CI 1.03–2.07, p = 0.03) adjusted for known risk factors for mortality including age, gender, FVC, DLCO, and honeycombing on HRCT (also shown in Fig. 2B). Patients with HP and honeycombing had a transplant free survival similar to IPF (median TFS 69.2 vs. 77.3 months, respectively, p = 0.68), whereas HP patients without honeycombing had a better TFS than IPF (median TFS 123.5 vs. 77.3 months, respectively, p = 0.003) (Fig. 3).

**Table 2:** Univariable and multivariable Cox model for transplant free survival in patients with fibrotic HP and IPF (N = 376, events = 163)

Variable	Univariable model		Multivariable model	
	HR (95% CI)	P value	HR (95% CI)	P value
AgeDx	1.015 (1.00-1.03)	0.068	1.027 (1.01–1.05)	0.0024
FemaleGender	0.75 (0.55–1.02)	0.07	0.67 (0.46–0.94)	0.026
Radiographic honeycombing	1.52 (1.13–2.05)	0.006	1.64 (1.20–2.26)	0.002
FVCperc	0.98 (0.97–0.99)	< 0.0001	0.99 (0.98-1.00)	0.048
DLCOperc	0.97 (0.96–0.98)	< 0.0001	0.97 (0.95–0.98)	< 0.0001
Never Smoker	0.91 (0.68–1.24)	0.58		
No antigen identified	1.38 (1.02–1.85)	0.036	1.03 (0.69–1.53)	0.9
Diagnostic category				
IPF	1.46 (1.02–2.15)	0.045	1.30 (0.84–2.03)	0.26
Moderate HP	1.11 (0.69–1.78)	0.66	0.80 (0.47–1.35)	0.41
High/definite HP	REF	REF	REF	REF

Data was then analyzed for each variable that is used in the determination of diagnostic confidence (antigen identification, HRCT pattern, and SLB pattern) adjusted for known predictors of mortality in HP, including age, gender, FVC, DLCO, and presence of honeycombing (Table 3). The diagnostic confidence was not entered in this model due to collinearity with HRCT pattern, antigen identification, and SLB pattern. The presence of honeycombing had a greater magnitude of impact on TFS than any factors in the diagnostic criteria for HP. Patients with identified inciting antigen had an improved survival compared to those without antigen in a univariable but not a multivariable model. Patients with a typical HP HRCT pattern had improved survival compared to those with a compatible scan or indeterminate scan in a univariable and multivariable model when patients without SLB were included; in a multivariable model that included only patients who had undergone SLB, the HRCT pattern was no longer significant (Table 3). SLB pattern was not associated with TFS in a univariable or multivariable mode, but radiographic honeycombing, FVC, and DLCO had a significant impact on TFS in a univariable and multivariable model of patients who underwent SLB.

**Table 3:** Univariable and multivariable Cox model for TFS in patients with fibrotic HP and IPF (N = 376, events = 163)

Variable	Univariable model		Multivariable model		Multivariable model of patients with SLB	
			(N = 376, events = 163)		(N = 178, events = 84)	
	HR (95% CI)	P value	HR (95% CI)	P value	HR (95% CI)	P value
AgeDx	1.015 (1.00-1.03)	0.068	1.027 (1.01–1.05)	0.0024	1.018 (0.99–1.04)	0.13
FemaleGender	0.75 (0.55–1.02)	0.07	0.77 (0.54–1.09)	0.15	0.71 (0.40–1.23)	0.23
No Identified Antigen	1.38 (1.02–1.85)	0.037	1.04 (0.73–1.47)	0.83	1.50 (0.90–2.51)	0.11
Compatible HP HRCT	1.337 (0.84–2.04)	0.20	1.27 (0.78–1.97)	0.32	1.34 (0.67–2.53)	0.38
Typical HP HRCT	0.54 (0.36–0.78)	0.0017	0.54 (0.34–0.86)	0.011	0.55 (0.28–1.02)	0.06
Honeycombing on HRCT	1.52 (1.13–2.05)	0.006	1.55 (1.125–2.14)	0.007	2.01 (1.23–3.29)	0.005
FVCperc	0.98 (0.97–0.99)	< 0.0001	0.99 (0.97-1.00)	0.033	0.98 (0.97-1.00)	0.012
DLCOperc	0.97 (0.96–0.98)	< 0.0001	0.97 (0.96–0.98)	< 0.0001	0.97 (0.95–0.99)	0.0008
Never Smoker	0.91 (0.68–1.24)	0.58				
BAL Lymph > 30%	0.79 (0.37–1.84)	0.56				
SLB indeterminate HP	1.65 (0.98–2.92)	0.07			1.23 (0.63–2.47)	
SLB compatible HP	1.42 (0.78–2.60)	0.25			1.20 (0.58–2.50)	

A Cox proportional hazard model was then constructed for patients with fibrotic HP of a moderate, high, or definite confidence, excluding patients with IPF, in order to be able to control for treatment with immunosuppression, which is routinely used in the treatment of HP but contraindicated in IPF. Male gender, honeycombing, and low DLCO had a reduced TFS in a univariable and multivariable analysis (Table 4). Among the 87 patients who received > 6 months of IS, 73 (80.4%) received mycophenolate, 22 (25.3%) received azathioprine, and 84 (96.6%) received prednisone. Sixty-one patients (70.1%) received both prednisone and mycophenolate in combination. Mycophenolate and azathioprine were not used simultaneously, but 12 patients (13.8%) were switched from one agent to the other due to side effects. The use of immunosuppression for > 6 months did not affect TFS in a univariable or multivariable model.

**Table 4:** Univariable and multivariable Cox model for TFS in fibrotic HP with moderate, high, or definite diagnostic confidence (N = 198, events = 71)

Variable	Univariable model		Multivariable model	
	HR (95% CI)	P value	HR (95% CI)	P value
AgeDx	1.015 (0.99–1.04)	0.22		
Female Gender	0.53 (0.32–0.85)	0.009	0.49 (0.28–0.85)	0.011
No Identified Antigen	1.30 (0.62–2.43)	0.45		
Compatible HP HRCT	0.69 (0.28–1.50)	0.37	1.39 (0.53–3.26)	0.47
Typical HP HRCT	0.47 (0.28–0.77)	0.003	0.79 (0.45–1.42)	0.43
Honeycombing on HRCT	1.8 (1.12–2.91)	0.014	1.78 (1.02–3.07)	0.041
FVCperc	0.98 (0.96–0.99)	0.005	0.99 (0.97–1.01)	0.31
DLCOperc	0.96 (0.94–0.98)	< 0.0001	0.96 (0.94–0.98)	< 0.0001
Never Smoker	1.05 (0.66–1.69)	0.84		
BAL Lymph > 30%	0.75 (0.30–2.02)	0.54		
SLB indeterminate HP	0.85 (0.28–2.14)	0.24		
SLB compatible HP	1.43 (0.79–2.64)	0.76		
Continuous IS	0.89 (0.54–1.43)	0.62	1.51 (0.86–2.60)	0.14

Compared to those without honeycombing, patients with honeycombing were more likely to be current or former smokers, had a lower DLCO at ILD diagnosis, had a higher degree of fibrosis on initial HRCT, were more likely to be treated with immunosuppression, and were more likely to have a secondary UIP pattern on SLB (Table 5).

**Table 5:** Characteristics of HP with HC vs HP without HC

Variable	HP with HC (N = 61)	HP without HC (N = 137)	P-value
Mean age (SD)	63.1 (11.3)	65.2 (9.3)	0.18
Male, No. (%)	34 (55.7)	65 (47.4)	0.36
Ethnicity, No. (%)			0.54
Non-Hispanic White	49 (80.3)	116 (84.7)	
Ever Smoker, N (%)	37 (60.7)	61 (44.5)	0.045
Antigen identified, No. (%)	55 (90.2)	117 (85.4)	0.49
Baseline Lung Function, mean (SD)			
FVC % predicted			
DLCO % predicted	64.3 (16.1)	67.3 (19.0)	0.28
	44.9 (14.2)	50.9 (18.2)	0.027
HRCT available for scoring	61 (100)	137 (100)	
Typical HP	27 (44.3)	89 (65.0)	0.38
Compatible HP	4 (6.6)	19 (13.9)	
Indeterminate HP	29 (47.5)	29 (21.2)	
HRCT UIP pattern			
HRCT definite UIP	10 (16.4)	0 (0)	0.27
HRCT probable UIP	0 (0)	5 (3.6)	
HRCT indeterminate UIP	3 (4.9)	13 (9.5)	
HRCT non-IPF	48 (78.7)	119 (86.9)	
Degree of fibrosis			0.001
Mild	10 (16.4)	77 (56.2)	
Moderate	30 (49.2)	49 (35.8)	
Severe	21 (34.4)	11 (8.0)	
Distribution of fibrosis			0.37
Lower lobe predominant	17 (27.9)	30 (21.9)	
Upper lobe predominant	25 (41.0)	52 (38.0)	
Diffuse	19 (31.1)	55 (40.1)	
BAL lymph performed	15 (24.6)	48 (35.0)	0.19
BAL lymph > 30%	5 (8.2)	19 (13.9)	0.77
SLB available for scoring	45 (73.8)	84 (61.3)	0.11
Typical HP	21 (34.4)	35 (25.5)	0.71
Compatible HP	19 (31.1)	40 (29.2)	
Indeterminate HP	5 (8.2)	9 (6.6)	
SLB secondary UIP pattern	33 (54.1)	42 (30.6)	0.015
Diagnostic confidence HP			0.65
Definite HP	28 (45.9)	57 (41.6)	
High probability HP	5 (8.2)	26 (19.0)	
Moderate probability HP	28 (45.9)	54 (39.4)	
Use of continuous immunosuppression	35 (57.4)	52 (38.0)	0.013
Death or transplant	18 (29.5)	40 (29.2)	1

To assess the impact of treatment with immunosuppression on TFS in fibrotic HP, a Cox model adjusted for age, gender, FVC % predicted, and DLCO % predicted was constructed (Table 6). There was a trend toward increased mortality in patients with radiographic honeycombing on continuous IS (HR 1.79, 95% CI 0.94–3.36, p = 0.07) compared to those with honeycombing without continuous IS (HR 1.09, 95% CI 0.44–2.72, p = 0.85), but p-interaction was 0.39, indicating that the effect of IS on TFS is not modified by the presence of HC. To avoid immortality bias, we ran the same analysis for HC and IS in patients who were ever exposed to MMF or AZA and p-interaction remained insignificant (p = 0.39). When patients on IS were evaluated in a Cox proportional hazard model, only DLCO was associated with TFS, with HC having a trend toward worsening mortality (HR 2.07, 95% CI 0.96–4.53, p = 0.066) (Table 7).

**Table 6:** Cox proportional hazards model for TFS in patients with HP adjusted for age, gender, FVC % predicted, and DLCO % predicted (N = 198, events = 71)

	N (events)	HR (95% CI)	p-value	p-interaction
HRCTHC (without considering treatment)	187 (64)	1.54 (0.93–2.54)	0.09	--
HRCTHC on IS	187 (64)	1.79 (0.94–3.36)	0.07	0.39
HRCTHC not on IS	187 (64)	1.09 (0.44–2.72)	0.85	

**Table 7:** Risk factors for death or transplant in patients with fibrotic HP on IS (N = 87, events = 38)

Variable	Univariable model		Multivariable model	
	HR (95% CI)	P value	HR (95% CI)	P value
AgeDx	1.02 (0.89–1.06)	0.25		
Female Gender	0.50 (0.27–0.93)	0.03	0.55 (0.25–1.22)	0.14
No Identified Antigen	0.66 (0.20–1.66)	0.44		
Compatible HP HRCT	1.69 (0.55–4.33)	1.03	2.12 (0.60–6.74)	0.21
Typical HP HRCT	0.47 (0.24–0.93)	0.027	0.91 (0.39–2.12)	0.82
Honeycombing on HRCT	1.95 (1.05–3.68)	0.035	2.07 (0.96–4.53)	0.066
FVCperc	0.99 (0.97-1.00)	0.14		
DLCOperc	0.96 (0.94–0.99)	0.002	0.96 (0.94–0.99)	0.005
Never Smoker	1.10 (0.60–2.06)	0.76		
BAL Lymph > 30%	0.46 (0.13–1.67)	0.22		
SLB indeterminate HP	0.76 (0.17–2.43)	0.68		
SLB compatible HP	1.55 (0.74–3.41)	0.25		

## Discussion

In this study, we showed that age, presence of honeycombing, and baseline FVC and DLCO are more important prognostic factors than the diagnostic confidence of HP. The presence of honeycombing has a greater impact on the TFS than the diagnosis of HP vs. IPF. Among the criteria used in the HP diagnostic guidelines [1], only typical HP scan impacted survival in a multivariable model, while identification of antigen and SLB findings had no impact on survival. Our IPF TFS of 77 months exceeds the previously reported 36–60 month survival rate. Finally, we demonstrated that the use of immunosuppression does not impact TFS in fibrotic HP, and while there is a trend toward worse survival on IS in those with HP with honeycombing, the presence of honeycombing does not modify response to IS in fibrotic HP.

**Fig. 4:**
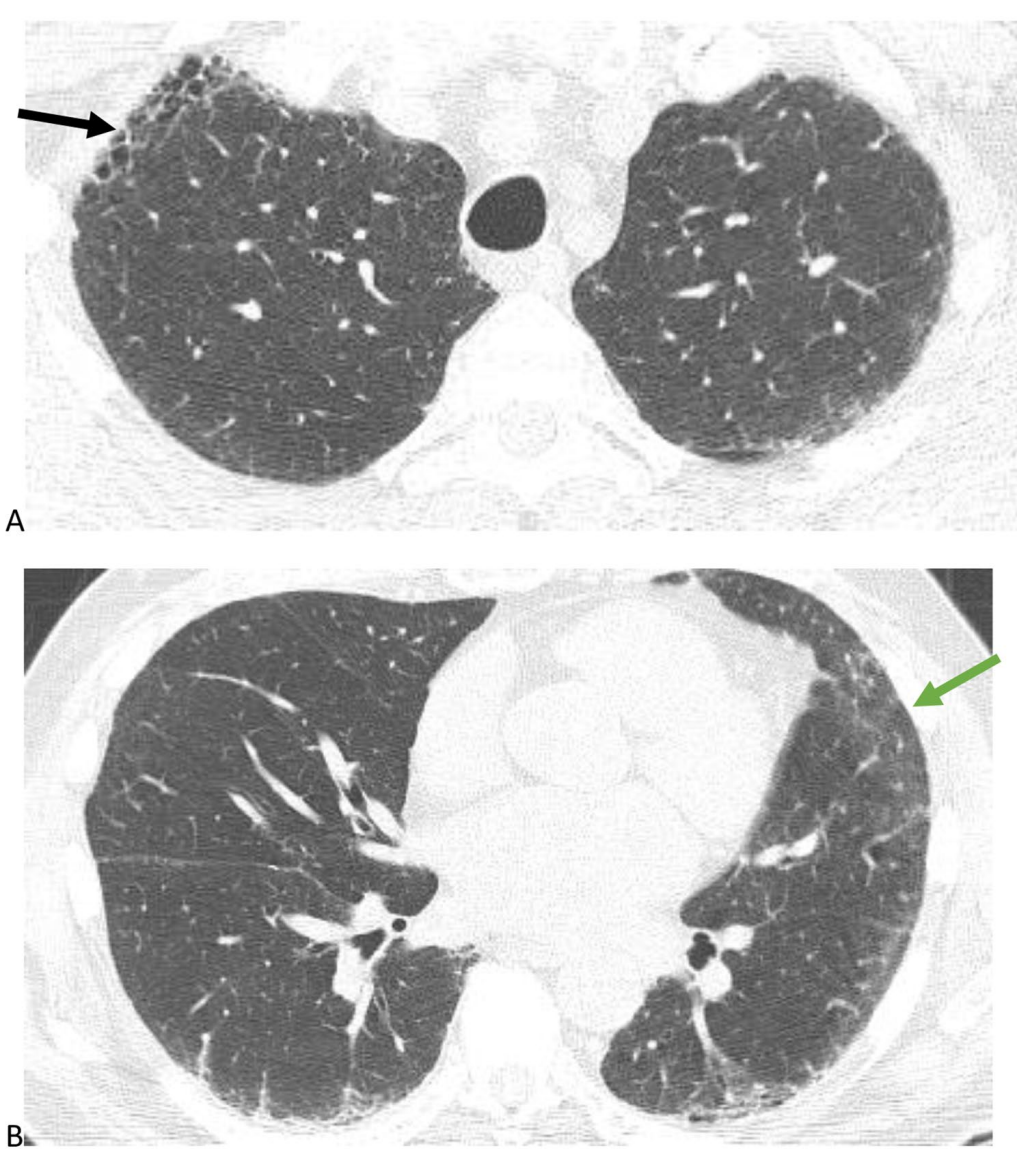
Minimal honeycombing (black arrow) in a patient with upper lobe predominant fibrosis (**4A)**, centrilobular nodules and air trapping, and ground glass (green arrow) suggestive of a typical hypersensitivity pneumonitis pattern (**4B**)

**Fig. 5:**
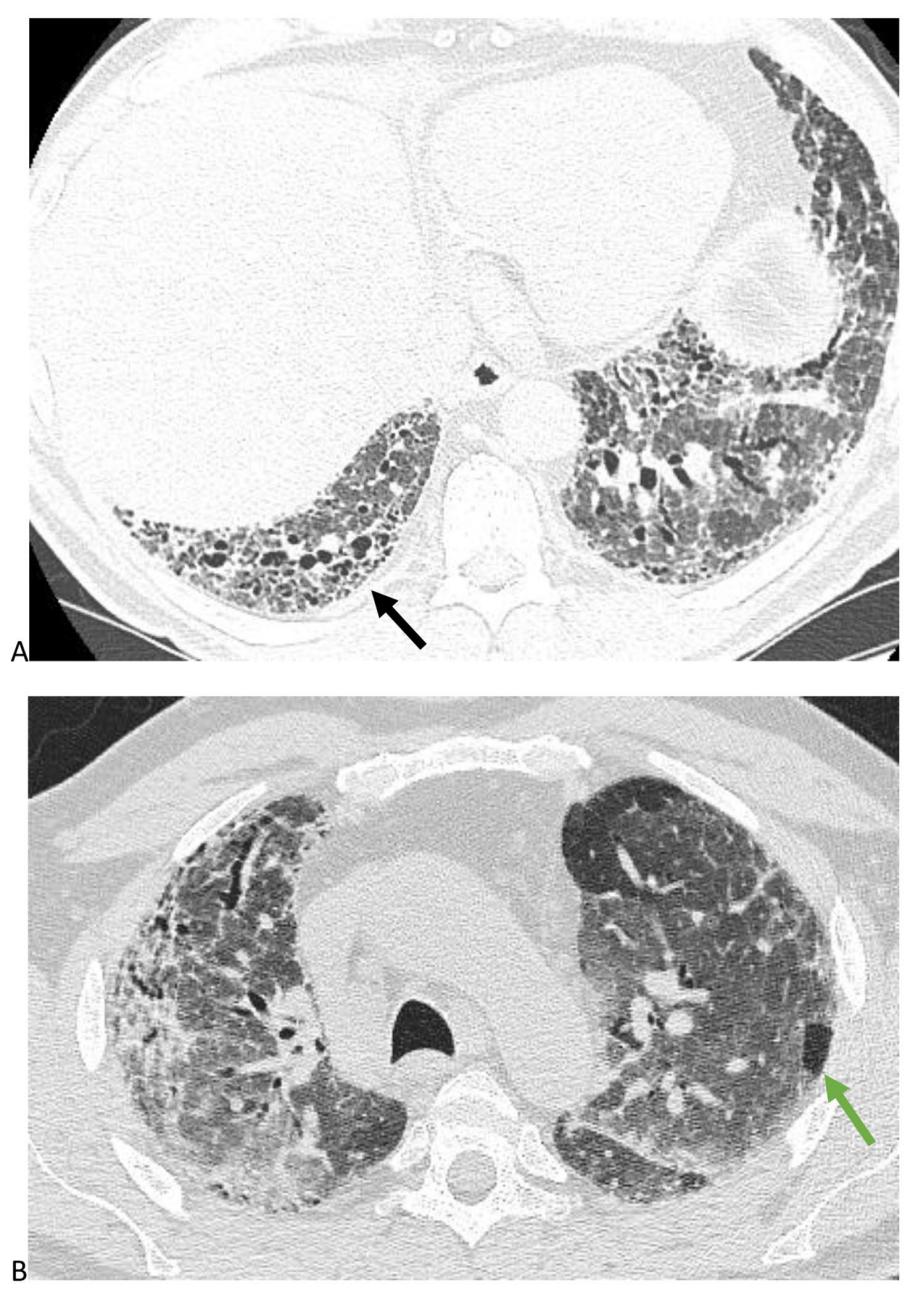
Pattern of basilar predominant reticulations, traction bronchiectasis, and honeycombing (black arrow) as well as 3 density sign (green arrow) (**5A**) and air trapping on expiratory images (green arrow) (**5B**) suggestive of a compatible hypersensitivity pneumonitis pattern

**Fig. 6:**
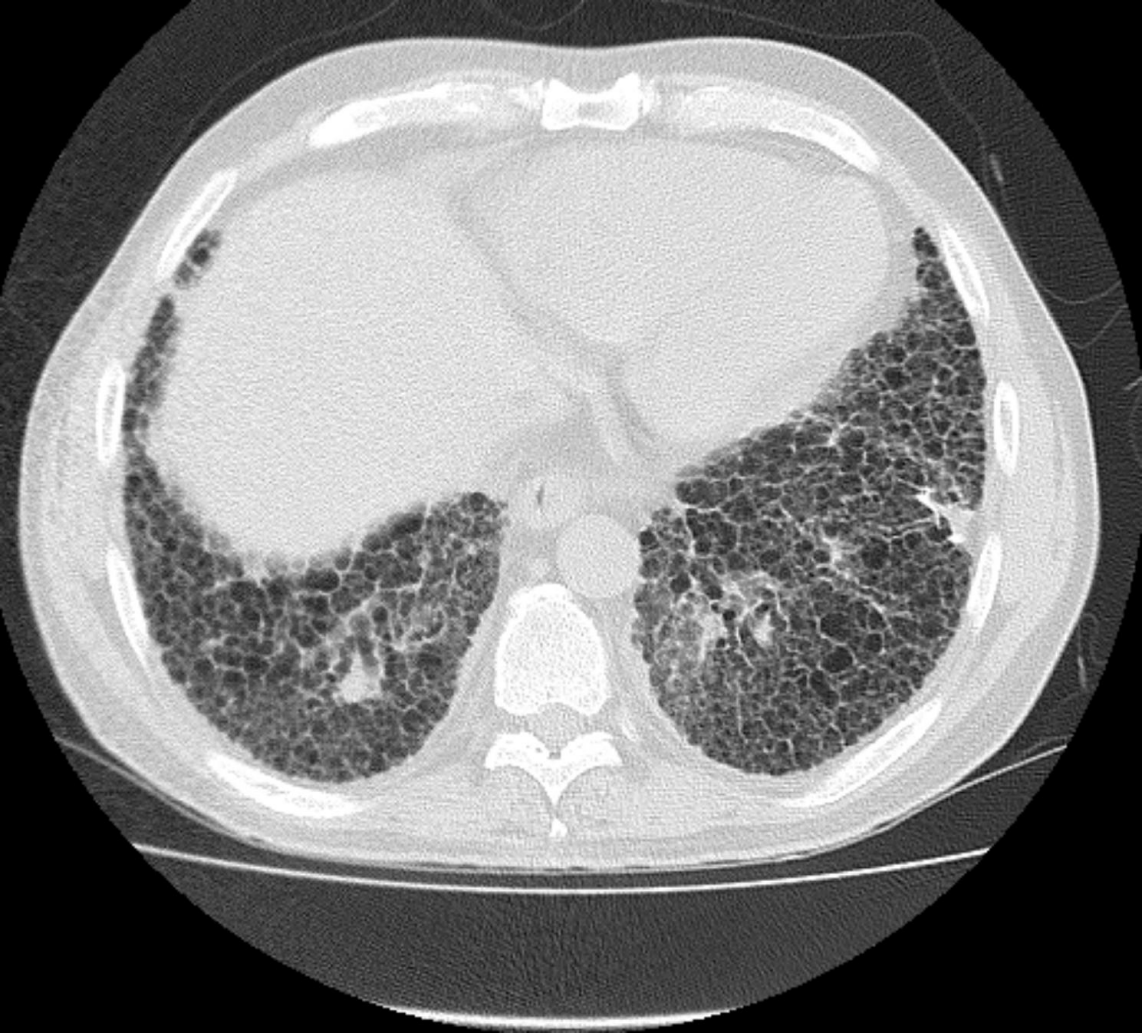
Typical usual interstitial pneumonia (UIP) pattern with basilar predominant honeycombing and absence of non-UIP features including ground glass or mosaicism suggestive of an indeterminate HP pattern

Studies have consistently shown that the presence of UIP pattern, which is characterized by honeycombing, is a negative prognostic factor in HP [3, 6]. This is the first study to our knowledge to compare the magnitude of the impact of UIP pattern on TFS relative to the diagnostic confidence of fibrotic HP in the latest iteration of the HP guidelines. We used the presence of honeycombing rather than definite UIP pattern as an independent variable because many patients with HP have air trapping that precludes classification of definite UIP pattern [4], making our sample size for definite UIP pattern small. Probable UIP pattern is not as predictive of histopathologic UIP in secondary UIP pattern as it is in IPF, so we did not use probable UIP pattern [9]. HRCTs with honeycombing can be classified as typical HP (Fig. 4), compatible HP (Fig. 5), or indeterminate HP (Fig. 6) depending on the degree of HC, location of HC, and presence of airway-centric findings and ground glass [1]. Because the presence of HC associated with reticulations and traction bronchiectasis signifies UIP pattern histopathologically and secondary UIP pattern has a similar prognosis compared to idiopathic UIP in non-HP diagnoses, we chose to use HC rather than definite UIP pattern as an independent variable, regardless of the location or degree of HC [7]. We did not have sufficient sample size to investigate the impact of the location and degree of HC, as the majority of patients had minimal basilar predominant HC.

Our results fit with prior data showing that across ILD diagnoses, patients with UIP pattern consistently have a lower TFS than patients with a non-UIP pattern [5, 7]. The poor survival in UIP pattern is likely explained by the overlap in environmental exposure, genetics, and rate of progression among patients with a UIP pattern and a non-IPF diagnosis compared to those with IPF. Environmental and host factors such as increasing age and smoking history are common between IPF and secondary UIP pattern [7]. Genetic variants associated with telomeres and transcriptosomal signatures are common to both IPF and secondary UIP pattern, including HP [10, 11]. Epithelial cell senescence and profibrotic subpopulations of cells are also seen during development in both IPF and HP with UIP [11]. Our data therefore supports the idea that HC of different etiologies may behave similarly and could be considered as a single diagnostic entity [7]. Our findings suggest that UIP pattern may be better considered as indeterminate for HP than compatible with HP, even with concomitant air trapping, in order to lessen the probability that patients with UIP pattern will be classified as HP.

While the use of IS in HP patients with HC did not meet statistical significance, there is a trend toward increased mortality in this population. Our findings fit with treatment with immunosuppression in IPF. In the PANTHER-IPF study, patients on immunosuppression had a higher mortality than those not treated with immunosuppression [12]. However, retrospective analysis taking into account telomere length suggest that the higher mortality in the IS group may have been driven by patients with short telomeres, with longer telomere length patients not having a change in survival after treatment with IS [13]. Our findings suggest that patients with HP with HC may fare worse and at a minimum overall do not have improved TFS with IS. This could suggest that early use of antifibrotics in this population rather than immunosuppression may be warranted. Our results, however, do not preclude response to IS in a subset of patients with HP and HC, and we were not able to control for indication bias. Prospective studies of the use of IS in this population are needed for evaluation.

Strengths of this study include use of published guidelines to classify patients as HP or IPF, which improves generalizability. Due to our large sample size, we were able to run a subset analysis on patients who had undergone SLB, which leads to more accurate classification and therefore improves generalizability. While an association between mortality and HC had been reported in fibrotic HP, we were able to evaluate the impact of HC on response to IS.

There are several limitations to the present study. Due to the retrospective nature of the study, we could not determine treatment adherence. In addition, we used clinical history to assess for environmental exposure rather than an industrial hygienist home assessment; this may limit accuracy of antigen detection but improves generalizability, as IH specialists are unavailable in many centers. Our results also suggest a better survival than prior studies of IPF, though this may reflect early detection and initiation of antifibrotics in our cohort. Our HP with HC cohort had a higher use of IS than HP without HC, which goes against our hypothesis that IS may worsen mortality in this population. We suggest that the increased use of IS in our cohort among patients with HP compared to those without is related to the severity of PFT impairment and higher severity of fibrosis in patients with HP with radiographic HC compared to those without. Because our cohort was enrolled before nintedanib was approved for the treatment of progressive fibrotic ILD [14]. IS was the only treatment option available for HP, and we are not able to effectively control for antifibrotic therapy added later in the treatment course after the majority of patients were censored. Finally, we used the presence of honeycombing as a characteristic in the multivariable model rather than definite UIP due to the small sample size of definite UIP pattern among those with HP, as the majority of HP patients had air trapping in > 3 lobes that precluded definite UIP classification.

In summary, our data suggests that UIP and baseline PFTs have a greater impact on TFS than the clinical diagnosis and that honeycombing is a predictor of poor TFS in fibrotic HP. Our data also suggests that IS does not impact TFS in HP even when adjusted for the presence of honeycombing. We suggest that invasive diagnostic testing including SLB may not be useful in predicting mortality in HP patients with UIP pattern and may potentially increase risk of immunosuppression.

## Electronic supplementary material

Below is the link to the electronic supplementary material.

Supplementary Material 1

## Data Availability

deidentified data is provided in the supplemental materials.

## Abbreviations

HP: Hypersensitivity pneumonitis
HRCT: High-resolution computed tomography
PFT: Pulmonary function test
FVC: Forced vital capacity
DLCO: Diffusing capacity for carbon monoxide
ILD: Interstitial lung disease
IPF: Idiopathic pulmonary fibrosis
HC: Honeycombing
BAL: Bronchoalveolar lavage
SLB: Surgical lung biopsy
UTSW: University of Texas Southwestern
TFS: Transplant free survival
HR: Hazard ratio
CI: Confidence interval
UIP: Usual interstitial pneumonia

## References

[1] Raghu G, Remy-Jardin M, Ryerson CJ, Myers JL, Kreuter M, Vasakova M (2020). Diagnosis of hypersensitivity pneumonitis in adults. An Official ATS/JRS/ALAT Clinical Practice Guideline. Am J Respir Crit Care Med.

[2] Fernandez Perez ER, Travis WD, Lynch DA, Brown KK, Johannson KA, Selman M (2021). Diagnosis and evaluation of hypersensitivity pneumonitis: CHEST Guideline and Expert Panel Report. Chest.

[3] Marinescu DC, Raghu G, Remy-Jardin M, Travis WD, Adegunsoye A, Beasley MB (2022). Integration and application of clinical practice guidelines for the diagnosis of idiopathic pulmonary fibrosis and fibrotic hypersensitivity pneumonitis. Chest.

[4] Raghu G, Remy-Jardin M, Richeldi L, Thomson CC, Inoue Y, Johkoh T (2022). Idiopathic pulmonary fibrosis (an update) and progressive pulmonary fibrosis in adults: an Official ATS/ERS/JRS/ALAT Clinical Practice Guideline. Am J Respir Crit Care Med.

[5] Adegunsoye A, Oldham JM, Bellam SK, Montner S, Churpek MM, Noth I (2019). Computed Tomography Honeycombing identifies a progressive fibrotic phenotype with increased mortality across diverse interstitial Lung Diseases. Ann Am Thorac Soc.

[6] Dasgupta S, Bhattacharya A, Abhijit RD, Roy Chowdhury S, Chaudhury K (2022). Risk factors associated with mortality in hypersensitivity pneumonitis: a meta-analysis. Expert Rev Respir Med.

[7] Selman M, Pardo A, Wells AU (2023). Usual interstitial pneumonia as a stand-alone diagnostic entity: the case for a paradigm shift?. Lancet Respir Med.

[8] Newton CA, Oldham JM, Ley B, Anand V, Adegunsoye A, Liu G et al. Telomere length and genetic variant associations with interstitial lung disease progression and survival. Eur Respir J. 2019;53(4). 10.1183/13993003.01641-2018 PMC6612265 30635297

[9] Tibana RCC, Soares MR, Storrer KM, de Souza Portes Meirelles G, Hidemi Nishiyama K, Missrie I (2020). Clinical diagnosis of patients subjected to surgical lung biopsy with a probable usual interstitial pneumonia pattern on high-resolution computed tomography. BMC Pulm Med.

[10] De Sadeleer LJ, McDonough JE, Schupp JC, Yan X, Vanstapel A, Van Herck A (2022). Lung microenvironments and Disease Progression in Fibrotic Hypersensitivity Pneumonitis. Am J Respir Crit Care Med.

[11] Lee JS, La J, Aziz S, Dobrinskikh E, Brownell R, Jones KD (2021). Molecular markers of telomere dysfunction and senescence are common findings in the usual interstitial pneumonia pattern of lung fibrosis. Histopathology.

[12] Research N, Raghu G, Anstrom KJ, King TE, Lasky JA, Martinez FJ, Idiopathic Pulmonary Fibrosis Clinical (2012). Prednisone, azathioprine, and N-acetylcysteine for pulmonary fibrosis. N Engl J Med.

[13] Newton CA, Zhang D, Oldham JM, Kozlitina J, Ma SF, Martinez FJ (2019). Telomere length and use of immunosuppressive medications in idiopathic pulmonary fibrosis. Am J Respir Crit Care Med.

[14] Flaherty KR, Wells AU, Cottin V, Devaraj A, Walsh SLF, Inoue Y (2019). Nintedanib in Progressive Fibrosing interstitial lung Diseases. N Engl J Med.

